# Motor Phenomena Associated With Leucine-Rich Glioma-Inactivated (LGI1) Emi-Encephalitis

**DOI:** 10.7759/cureus.35882

**Published:** 2023-03-07

**Authors:** Maurizio Giorelli, Maria S Aniello, Sergio Altomare, Ruggiero Leone, Daniele Liuzzi

**Affiliations:** 1 Operative Unit of Neurology, "Dimiccoli" General Hospital, Barletta, ITA

**Keywords:** lgi1 antibody autoimmune encephalitis, fbds, temporal lobe, caudatum, epilptetic spasms, chorea

## Abstract

LGI1 encephalitis is a rare immune-mediated brain disorder. Its typical features include faciobrachial dystonic seizures (FBDS), startle reactions, chorea, myoclonus, atypical parkinsonism, cogni­tive impairment, and personality changes.

We report the case of a 57-year-old woman presenting with distinct patterns of involuntary movements, including faciobrachial dystonic spasms, dyskinetic movements, and chorea. Magnetic resonance imaging (MRI) and tests on blood and cerebrospinal fluid (CSF) demonstrated encephalitis involving the right temporal lobe and caudate nucleus and associated with LGI1-antibody.

LGI1 encephalitis may present with simultaneous distinct patterns of movement disorders depending on the cortical and subcortical structures involved in the disease.

## Introduction

Leucine-rich glioma inactivated (LGI1) encephalitis is an autoimmune disorder associated with antibodies against cell surface antigens, usually leading to rapidly progressive cortical and subcortical cognitive impairment, hyponatremia, and movement disorders [1]. A plethora of involuntary movements have been described in LGI1-encephalitis, including faciobrachial dystonic seizures (FBDS), chorea, myoclonus, parkinsonism, and paroxysmal dyskinesia [2]. The pathogenesis of FBDS is attributed to either epileptic spasms [3] or true dystonic contractions [4]. Intriguingly, LGI1 encephalitis may involve the basal ganglia and temporal lobes in an associated or isolated fashion [5].

## Case presentation

A 57-year-old woman presented to the emergency department (ED) complaining of memory impairment, recurrent and transitory episodes of strength reduction, rigidity, and involuntary movements of her left limbs, which had progressed from the arm to the ipsilateral leg in the preceding week. Her medical history was relevant only for fluctuating psoriatic dermatitis that was episodically treated with topical corticosteroids. On admission, the patient presented with slight hyponatremia (132.8 mEq/l). The results of all other blood tests were normal. Although she appeared slightly perplexed and hypomimetic and responded to all questions with latency, the Frontal Assessment Battery and Mini-Mental State Examination did not reveal any significant deficits in either executive functions or temporal-spatial orientation, explicit and verbal memory, or motor praxis. The observation showed different patterns of involuntary movements (Video 1). Written consent from the patient to reveal their identity in this article is provided in the appendix.

**Video 1:** Motor phenomena in our patient Motor phenomena associated with leucine-rich glioma-inactivated (LGI1) emi-encephalitis

First, stereotyped, unilateral, single-shot, violent spasms (lasting up to two to three seconds) consisting of flexion and rotation of the head on the neck and to the left side, flexion of the left arm at the elbow, and adduction to the trunk, both immediately followed by the inward rotation of the ipsilateral leg and associated with staring and speech arrest. The episodes were preceded by emotional discomfort followed by intense sweating. Low-amplitude spikes were recorded from contralateral, right, and temporal electrodes on the electroencephalogram (EEG) and preceded muscle contractions of the left biceps, left ulnaris flexor carpi, and left abductor allucis as recorded by the electromyogram with a latency ranging up to one second. These episodes were compatible with FBDS of epileptic origin. Second, the elevation of the right arm and twisting of the fingers was performed while performing a simple motor task with the contralateral arm (touching the right ear with the left hand). This movement emerged when the right limb was relaxed, and the left limb was activated, possibly representing focal dyskinesias as a consequence of the failure of cortical-subcortical inhibitory circuits and, therefore, motor overflow. Third, choreic movements of the right fingers, especially when distracted by mental tasks, are followed by FBDS.

**Figure 1:**
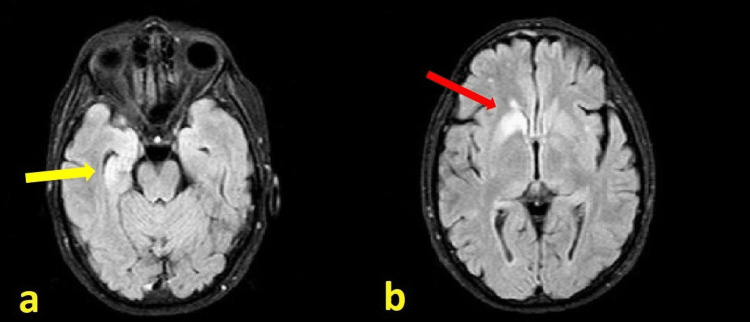
Magnetic resonance imaging of the brain MRI fluid-attenuated inversion recovery (FLAIR) showing hyperintensity in the medial right temporal lobe (a; yellow arrow) and anterior right caudatus (b; red arrow).

Magnetic resonance imaging (MRI) revealed hyperintensity of the right temporal lobe as well as of the right caudate nucleus in sequences acquired according to the long relaxation of protons (T2/FLAIR; Figure 1).

We suspected encephalitis and performed extensive blood and cerebrospinal fluid (CSF) examinations. Tests included rheumatologic, hormonal, infectious, and neoplastic markers as well as antibodies known to be reactive against neurons. Anti-LGI1 antibodies were reactive in both serum and CSF. Polymerase chain reaction for severe acute respiratory syndrome coronavirus-2 was negative, and CSF standards and markers of neurodegeneration were normal. Computed tomography of the neck, chest, abdomen, and pelvis did not reveal any underlying tumours. The patient underwent five days of intravenous pulse steroids (1000 mg of methylprednisolone per day) and three complete cycles of alternate-day double-filtration aphaeretic treatment (filter 3a, Evaflux^TM^; Kawasumi Laboratories, Tokyo, Japan). Soon after the third administration of methylprednisolone, the patient began to recover her emotional participation in the surrounding environment, which progressively improved to full resolution following the completion of the aphaeretic schedule. Similarly, the pathological movements clearly began to decrease at the end of the corticosteroid pulse, progressively subsided, and completely disappeared within a span of two weeks. At the nine-month follow-up, the patient had recovered completely. Brain MRI was completely normal, and no pathological signs or symptoms were noted.

## Discussion

Motor phenomena associated with anti-LGI1 encephalitis in this case included hyperkinetic FBDS, which might be related to the observed MRI abnormalities in both the right caudate nucleus and temporal lobe [5]. FBDS are usually characterised by unilateral, shock-like, or slow-driven dystonic posturing and jerks of the upper limb and face. Besides their ictal presentation, the pathogenesis remains a matter of debate due to both epileptic cortical discharges and normal EEG, suggesting true dystonic contractions [4]. Our patient presented with symptoms resembling a temporal seizure (suspension of consciousness preceded by emotional discomfort followed by sweating). EEG monitoring confirmed neuronal discharges in the right temporal lobe, supporting the epileptic pathogenesis of this phenomenon. FBDS was first described as pathognomonic of anti-LGI-associated encephalitis [1]. Indeed, observation of these movements in our patient immediately prompted us to investigate immune-mediated encephalitis associated with antibodies directed against antigens on the neuronal surface, including voltage- or neurotransmitter-gated channels.

Moreover, the patient in this case showed choreic movements ipsilateral to the hemisphere involved, which is a rare phenomenon in brain lesions, and its underlying mechanism is still poorly understood. We speculated that the presence of lesions in both the cortex and basal ganglia in the patient may have caused a loss of inhibitory control exerted by cortical neurones on the ipsilateral subcortical structures. Although sustained by a different disease, namely cerebral infarction, ipsilateral chorea has already been documented [6]. Reduced subthalamic-driven activation of the inhibitory internal globus pallidus and ipsilateral chorea may have been caused by underactivity of the hyperdirect route.

Despite the absence of MRI-detectable brain lesions, it has been previously described that chorea in LGI1-encephalitis may affect one side of the body [7]. It is reasonable to think that microscopic lesions or neuron-specific dysregulation in isolated cell populations might be present in the apparently unaffected basal ganglia and underlie the generation of contralateral chorea in our patient. Our findings are in line with a previous paper that described substantial asymmetry in anti-LGI1 encephalitis [5]. In contrast to post-mortem studies showing that the expression of the LG1-1 molecule is preferential in the left basal ganglia [8], right brain structures were mostly detected by MRI in our patient. Although the recognition of FBDS is critical to suspecting anti-LGI1-associated encephalitis, this specific pattern of involuntary movements may be absent in some patients [7].

## Conclusions

Although FBDS are pathognomic of anti-LGI1 encephalitis, the sudden onset of involuntary movements should prompt clinicians to recommend a wide panel of investigations, including investigating for anti-LGI1 antibodies, even in the absence of MRI-detectable brain lesions. Anti-LGI1 encephalitis is rarely associated with malignant neoplasms. Immunotherapy may lead to full resolution of the clinical syndrome, as in the case of our patient.
